# Impact of additional mattresses in emergency CT on the automated patient centering proposed by a 3D camera: a phantom study

**DOI:** 10.1038/s41598-021-92637-7

**Published:** 2021-06-23

**Authors:** Joël Greffier, Julien Frandon, Hélène de Forges, Aymeric Hamard, Asmaa Belaouni, Jean Baptiste Wahl, Djamel Dabli, Jean Paul Beregi

**Affiliations:** 1 Department of Medical Imaging, CHU Nimes, Univ Montpellier, Medical Imaging Group Nimes, EA2992, Bd Prof Robert Debré, 30029 Nîmes Cedex 9, France; 2 GE Healthcare PIA, Strasbourg, France

**Keywords:** Applied physics, Techniques and instrumentation

## Abstract

To assess the impact of the use of additional mattresses of different thicknesses on radiation dose and image noise based on the patient centering proposed by a 3D camera for CT. An anthropomorphic phantom was placed on mattresses of different thicknesses (from 3.5 to 13.5 cm) on the table of a CT scanner. The automated patient centering proposed by a 3D camera was analysed as a function of mattress thickness and corrected for table height. For this purpose, the impact on image noise in the lung tissues in the chest area and in the soft tissues in the abdomen-pelvis area, modulated mAs (mAs_mod_) by the tube current modulation system (TCM) and volume CT dose index (CTDI_vol_) was assessed slice-by-slice along the z-axis after CT scans. With the use of a mattress, the automated centering proposed by the 3D camera resulted in placement of the phantom above the isocentre. This incorrect positioning led to a significant increase in the mAs_mod_ along the z-axis (p < 0.05) and in the CTDI_vol_. Image noise was significantly higher (p < 0.05) for automated phantom centering than with manual phantom centering. Differences of image noise between acquisitions with mattresses after automatic and manual phantom centering increased with the mattress thicknesses. The use of an additional mattress placed between the patient’s back and the table-top would require correcting the vertical centering proposed by the 3D camera. This manual correction is essential to avoid increased dose delivered to the patient and higher image noise.

## Introduction

During a CT scan examination, centering the patient is an essential step in ensuring a quality examination. Indeed, for many years, all CT scans have been equipped with tube current modulation systems (TCMs)^1–4^. These systems adapt the tube current (mAs) according to the patient's attenuation. For this system to be effective, the patient must be centered at the isocentre of the CT scan. When the patient is centered, his or her attenuation can be correctly measured on the topographic images and during the CT examination, and the mAs used can be adapted to the patient’s morphology. This guarantees uniform image quality throughout the entire acquisition in the longitudinal axis and reduces the number of artefacts^1–5^. In addition, centering the patient at the isocentre is also essential to ensure optimal functioning of the bowtie filter. The bowtie filter allows correction of the attenuation differences between the centre and the periphery of the patient to homogenize the signal received by the CT detectors. If the patient is off-centered, the bowtie filter correction would be non-uniform, resulting in an increase in the image noise and an incorrect dose distribution for the patient^6.^

Recently, 3D cameras for body contour detection have been installed on CT scans from two manufacturers (Siemens Healthineers and GE Healthcare)^7^. These 3D cameras, positioned above the patient and connected to the CT scan, allow the patient to be centered both on the anatomical area to be examined (longitudinal position) and on the isocentre (vertical position). The purpose of these 3D cameras is to help the operator in the correct centering of the patient and allow standardization of patient centering among operators to improve the practice.

Operation of the 3D camera developed by Siemens Healthineers has been detailed by R. Booij et al.^7^. This 3D camera is equipped with an infrared light source and sensor and a visible light camera. It uses an artificial intelligence algorithm that defines the patient's isocentre and the area to be radiographed according to the protocol selected. To do this, the camera takes an image of the patient positioned on the examination table to detect the patient and the body regions. The system then proposes the longitudinal range of acquisition based on the selected protocol and the detected body regions on the patient image. Then, the camera quickly and simplistically defines a fallback isocentre for the patient by determining the isocentre of the patient from the distance between the table top and the patient top over the entire area to be radiographed. Finally, the isocentre is adjusted from an avatar, which is a statistical shape model learned from a training database. In all cases, the radiographer can manually correct the centering of the patient in the longitudinal and vertical directions.

Initial studies on adult and paediatric patients have shown that the 3D camera developed by Siemens Healthineers allows precise positioning of the patient, limiting the variability of centering between radiographers^7–9^. However, the authors also concluded that the patient's centering could sometimes be corrected by the radiographer when the system fails or in challenging cases^7^. These studies used the 3D camera workflow where the patients were placed on the CT table using the classical manufacturer mattress, as the 3D camera has only been calibrated and approved for this mattress. However, it is not known what impact the use of an additional mattress between the patient's back and the table top, corresponding to an uncalibrated, off-label use of the 3D camera, has on defining the patient's isocentre. This is a relevant question, as in emergency departments, patients are very often unable to move and are transferred from their stretcher to the CT scan table with their mattress to reduce pain.

The purpose of our study is therefore to assess the impact of the use of additional mattresses of different thicknesses on the patient centering proposed by the 3D camera. For this, the influence on the radiation dose and on image noise was assessed using automatic slice-by-slice image analysis software.

## Materials and methods

### CT scan and CareDose-4D

Acquisitions were performed on the third generation of the dual-source CT system SOMATOM Force (Siemens Healthineers). This CT scanner was equipped with a 3D camera body contour detection system and a CareDose 4D TCM system, which uses both angular and longitudinal modulations^1,2^. Longitudinal modulation adjusts the mAs along the longitudinal axis (z-axis) based on the patient attenuation determined from topographic images. Angular modulation (x–y plane) adjusts the mAs during rotation of the tube around the patient with a delay of a half rotation. Five modulation strengths can be used with the CareDose 4D system to optimise the image quality and the dose delivered as function of the patient morphology and attenuation.

### Phantom and mattresses

A CT Torso CTU-41 anthropomorphic phantom (Kyoto Kagaku) was used to simulate a patient. The phantom has internal structures corresponding to patient tissues and simulates a patient with a body mass index close to 18 kg/m^2^. To assess the attenuation of the chest, abdomen and pelvis, the phantom was scanned from the last cervical vertebra to the pubic symphysis, which corresponds to an explored length of approximately 60 cm.

The phantom was placed on different mattresses positioned on the table top. A fixed mattress (approximately 2.5 cm thick) usually used for all patients in clinical practice was fixed on the table top and used for all acquisitions. Different additional mattresses positioned between the phantom’s back and the fixed mattress were used: a 3.5 cm thick mattress (grey), a 10 cm thick mattress (orange) and a 13.5 cm thick mattress (grey and orange).

### Acquisition and reconstruction protocols

Acquisitions of the phantom with or without an additional mattress was performed using identical acquisition parameters: 120 kVp, quality reference mAs of 240, pitch factor of 0.6, beam collimation of 96 × 0.6 mm and rotation time of 0.5 s.

For each acquisition, the 3D camera and CareDose 4D system with the “Average” modulation strength were used. Images were first acquired by using the height centering automatically proposed by the 3D camera. A second acquisition was performed when the phantom was placed on one of the additional mattresses after manually adjusting the height of the table to place the phantom at the isocentre. To ensure that the phantom was manually centered at the same height, a marker was positioned on the phantom.

Raw data were reconstructed using level 3 of the advanced modeled iterative reconstruction algorithm (ADMIRE) with the reconstruction kernel ‘‘Regular Sharpness’’ level 40 (Br40, for soft tissue exploration). Images were reconstructed using a slice thickness close to 1 mm (0.7 mm increment) and a field of view of 500 mm.

### Deviation of patient centering

The phantom centering was assessed in two different ways. First, the table height was retrieved from the CT gantry for all acquisitions. The table height for each acquisition with the use of an additional mattress was compared to that of the reference acquisition without the additional mattress, and the centering deviation was computed. Second, we used a beta version of a tool available in our Radiation Dose Management System (RDMS) DoseWatch (GE Healthcare) and validated by our institution. This tool defines on each slice the centre of the patient body as the barycentre of the pixels. The contour of the patient body is determined using an Otsu threshold algorithm. Then, the centre of the patient body is compared with the centre of the image and the tool gives the variation along both axes, x and y.

**Figure 1:**
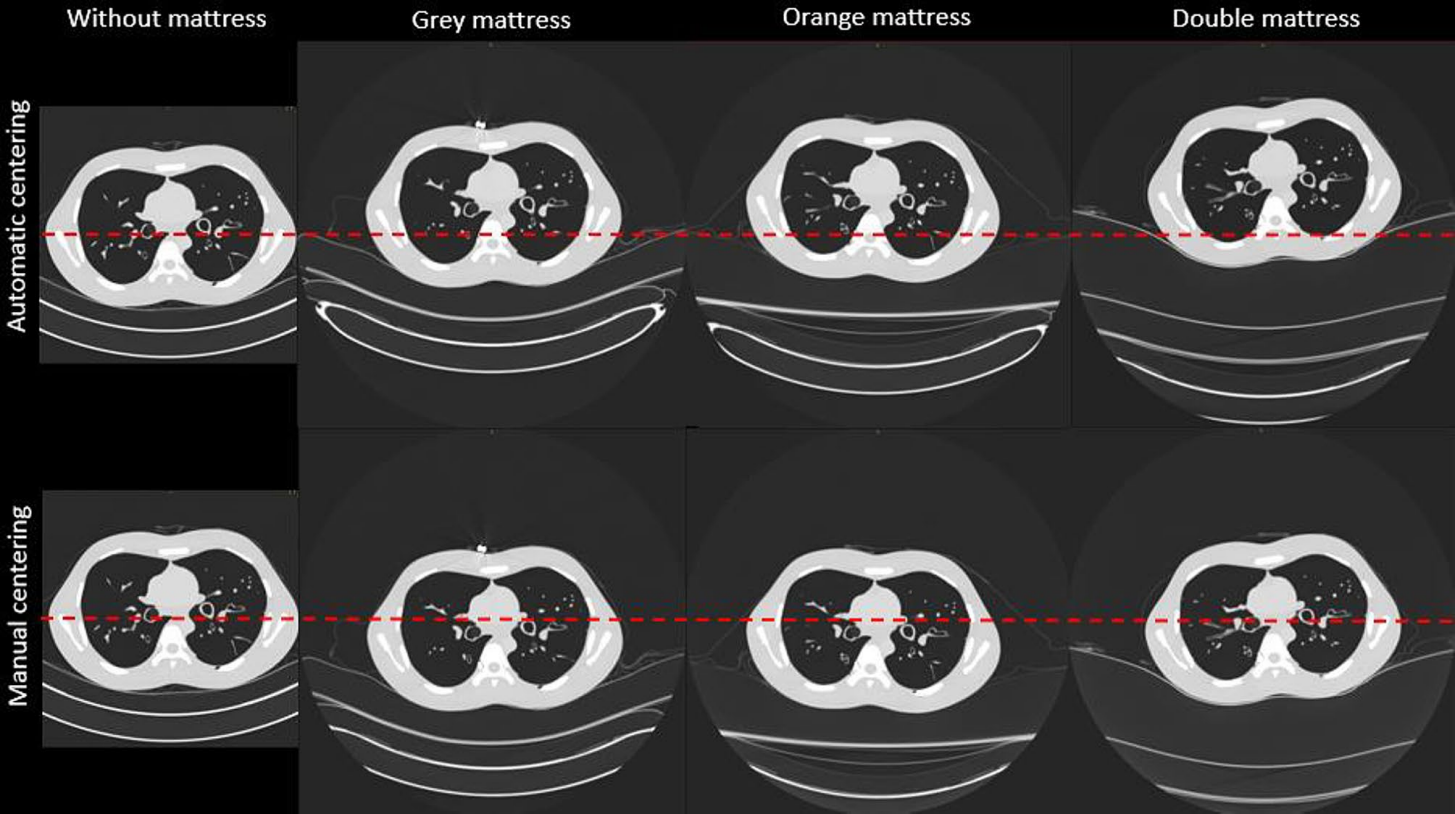
Images of the phantom at the level of the thorax after centering the phantom automatically with the 3D camera and then manually without and with the three additional mattresses (window level: − 500 HU and width: 1500 HU). The red dotted line represents the centre of the field of view.

In addition, to assess both the compression of the mattress and the space between the fixed and additional mattress, which impacted the camera centering algorithm, the distance between the phantom’s back and the top of the fixed mattress on the images of the phantom was also measured. This measurement was performed manually for each phantom on the same 20 slices along the z-axis on a SyngoVia workstation (Fig. 1).

**Figure 2:**
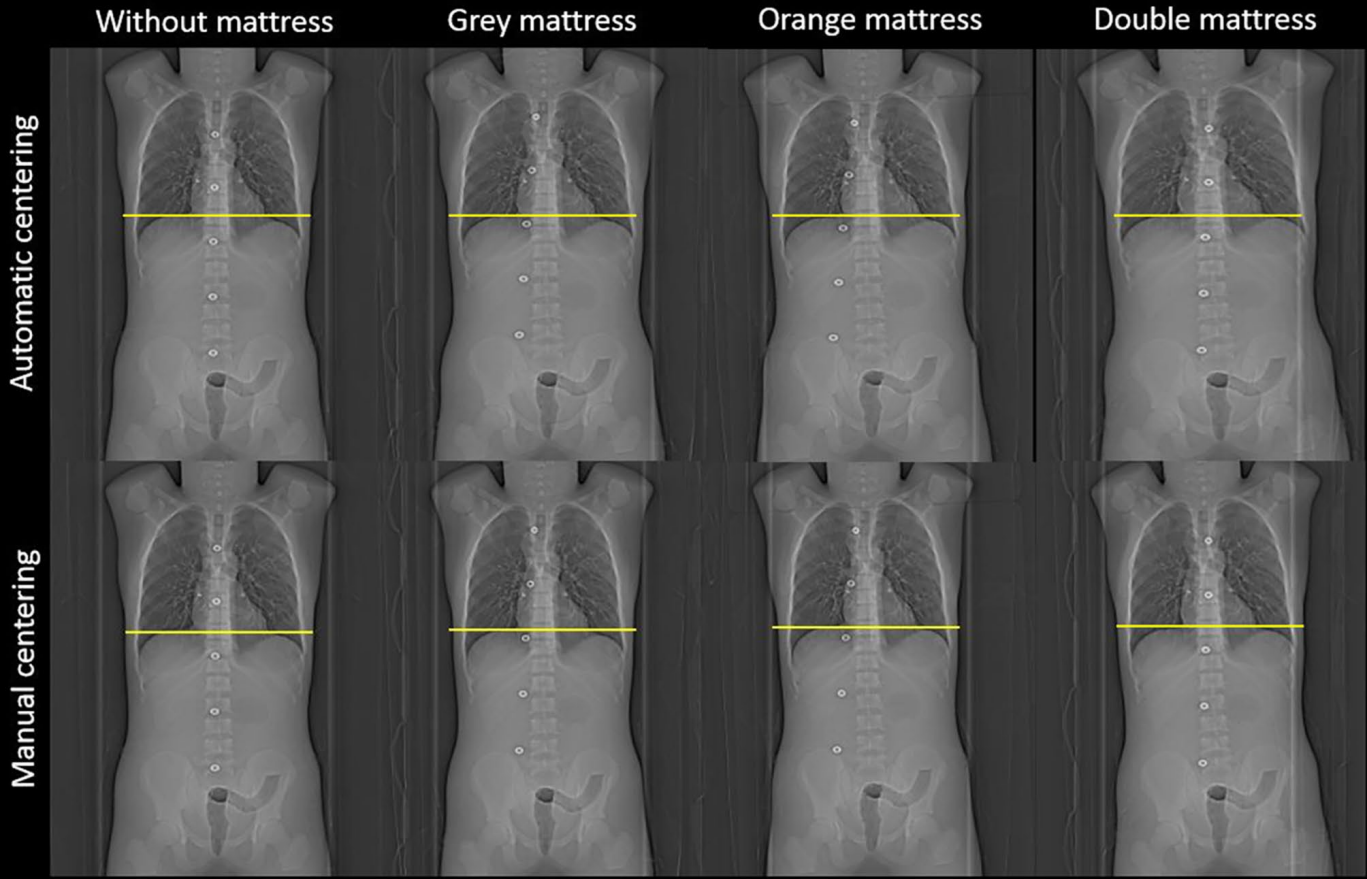
Topographic images obtained after centering the phantom automatically with the 3D camera then manually without and with the three additional mattresses. The yellow line corresponds to the phantom width from the image acquisition without an additional mattress.

Last, to assess the impact of phantom centering on the topographic images, the phantom width was measured on the topographic images by drawing a line passing through the centre of the 10th thoracic vertebra (Fig. 2).

### mAs and image noise per slice along the z-axis

For each acquisition, the modulated mAs (mAs_mod_) per slice was extracted from DICOM header files by our RDMS.

Image noise per slice was calculated using a beta version of a tool available in our RDMS and validated by our institution. For each slice, this tool first computes the noise map of the image, representing the local noise for each pixel, calculated using a sliding window.

Considering the image $\left({I}_{i,j}\right)$ with the size *n* × *m*, the tool defines the regions of interest (bones, soft tissues, lungs) by segmenting the image using the Hounsfield reference scale. For each region of interest k, the tool defines I^k^ ⊂ *I* the set of pixels belonging in this region.

The noise map $\left({N}_{i,j}^{k}\right)$ is calculated as

$${N}_{i,j}^{k}={\sigma }_{{\mathrm{\Delta }}_{i,j}^{\mathrm{k}}}\left({\mathrm{I}}_{i,j}^{k}\right)$$

where ${\sigma }_{{\mathrm{\Delta }}_{i,j}^{\mathrm{k}}}$ is the standard deviation evaluated on the neighborhood ${\mathrm{\Delta }}_{i,j}^{\mathrm{k}}$ of the pixel ${\mathrm{I}}_{i,j}^{k}$. In practice we used a 10 pixels window centered on the pixel ${I}_{i,j}$:

$$\Delta _{{i,j}}^{{\text{k}}} = \left\{ {{\text{I}}_{{p,q}}^{{\text{k}}} ,~i - 5 \le p \le i + 5,~j - 5 \le q \le j + 5} \right\}$$

The global noise map (N_﻿i,j_) is then computed as the union of the noise map per region, $N={\cup }_{k}{N}^{k}$.

Using the noise map per region prevents from an over-estimated quantity of noise in the boundary areas between two regions of interest (two kinds of tissue). For instance, when the sliding window is simultaneously on pixels representing air and pixels representing soft tissues. In such region, the local noise will be systematically higher due to the important gap between the characteristic amplitudes of these two types of tissue.

In addition, the global noise level can be evaluated as the mean value of the noise map:

$$\nu \left(I\right)=E\left({N}_{i,j}\right)$$

The computation of the noise map is only performed on pixels representing the patient body. A contouring algorithm is used to determine a mask applied before the creation of the regions of interest I^k^. The contour detection is made using an Otsu threshold algorithm.

In this study, we extracted the image noise measured in the lung ROI for each slice containing lung tissues in the chest area and in the soft tissues ROI for each slice containing the soft tissues in the abdomen-pelvis area.

Image noise of soft tissues and lungs and the mAs_mod_ along the z-axis were traced for each acquisition to compare the impact on image quality and dose delivered to the patient of both the manual and automatic patient centering using additional mattress.

### Dosimetry

For each acquisition, the volume CT dose index (CTDI_vol_), determined for a 32 cm diameter reference phantom, was recovered on the RDMS at the end of the CT acquisitions.

### Statistical analysis

Statistical analyses were carried out using XLSTAT software (Microsoft Excel). Comparisons of mAs_mod_ and image noise between acquisitions performed with and without additional mattresses were carried out using paired Student’s t-test. A p-value lower than 0.05 was considered significant.

## Results

### Deviation of patient centering

Compared to the acquisition without an additional mattress, considered hereafter as the reference acquisition, the 3D camera proposed lowering the table by 16 mm for the grey mattress, 44 mm for the orange mattress and 84.5 mm for the double mattress (Table 1). The distances between the phantom centre and the image centre were higher than in the reference acquisition by 8 mm, 41 mm and 58 mm, respectively.

**Table 1:** Table height, distance between the phantom and image centres, distance between the phantom and the table top and phantom width obtained for acquisitions of phantom with and without an additional mattress.

Mattress	Patient centering	Table height (mm)	Distance between phantom and image centres (mm)	Distance between phantom and table top (mm)	Phantom width (cm)
Without	Automatic	120	11 ± 7	–	309
Grey (3.5 cm)	Automatic	136	39 ± 11	33 ± 5	327
	Manual	183.5	12 ± 8		299
Orange (10 cm)	Automatic	164	50 ± 13	103 ± 14	332
	Manual	220	13 ± 8		299
Double (13.5 cm)	Automatic	204.5	71 ± 12	163 ± 20	344
	Manual	268	15 ± 8		307

Figure 1 depicts the differences in the automatic and manual centering of the phantom according to the mattress used. A thicker mattress resulted in a greater height for the centre of the phantom compared to the centre of the image. After correcting the patient centering, the table height was reduced to 47.5 mm, 56 mm and 64 mm reducing the distance between phantom and image centres. After these corrections, the phantom centering was similar for all acquisitions (± 10 mm) (Table 1).

The mean distance between the phantom’s back and the table top was 33 ± 5 mm for the grey mattress, 103 ± 14 mm for the orange mattress and 163 ± 20 mm for the double mattress. This distance takes into account the mattress compression by the phantom and the air space between the mattress and the table top present with the orange and double mattresses (Fig. 1).

Figure 2 depicts the topographic images of the phantom obtained as a function of the automatic or manual phantom centering for each mattress. The height positioning of the phantom with the automatic phantom centering leads to an increase in the width of the phantom on topographic image (Table 1). Compared with that of the reference acquisition, the width was increased by 18 mm, 23 mm and 35 mm for the grey, orange and double mattresses, respectively. After manually correcting the phantom centering, the width was similar for all acquisitions (302 ± 5 mm).

### mAs and image noise per slice along the z-axis

**Figure 3:**
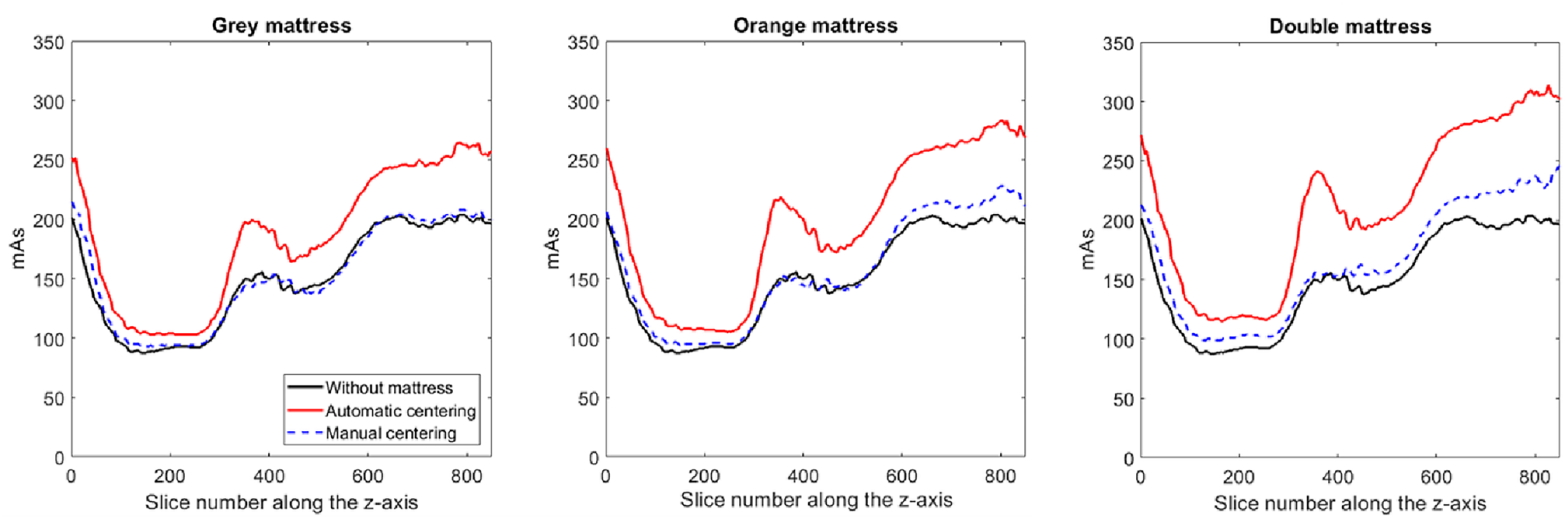
Modulated mAs along the z-axis obtained for image acquisitions without and with additional mattresses after automatic and manual phantom centering. The differences in mAs values along the z axis are related to the differences in anatomical structures in the phantom (shoulder, chest, abdomen, pelvis).

Figure 3 depicts the modulated mAs along the z-axis for each acquisition. The lowest values of mAs were found in the middle of the chest area, and the highest values were found in the shoulder and pelvis areas.

Compared with that of the reference acquisitions, mAs_mod_ was significantly higher for the acquisitions from each of the mattresses used (p < 0.05) (Table 2). The average differences with the reference acquisition were 23% ± 6% for the grey mattress, 29% ± 8% for the orange mattress and 41% ± 9% for the double mattress. The CTDI_vol_ being directly proportional to the mAs, these increases in mAs increased the CTDI_vol_ to 23%, 31% and 41%, respectively (Table 2). After correction, the mean mAs_mod_ was slightly higher than that of the reference acquisition by 1% ± 3%, 5% ± 4% and 11% ± 6%, respectively, and the difference was significant only for the double mattress (p < 0.05). Additionally, after correction, similar CTDI_vol_ values were obtained between the reference and grey mattress acquisitions, but the CTDI_vol_ increased by 4% and 12% for the orange and double mattresses, respectively (Table 2).

**Table 2:** Volume CT dose index and mean modulated mAs obtained for acquisitions of phantom with and without an additional mattress.

Mattress	Patient centering	CTDI_vol_ (mGy)	mAs_mod_
Without	Automatic	8.68	150 ± 41
Grey (3.5 cm)	Automatic	10.66	186 ± 56
	Manual	8.71	153 ± 42
Orange (10 cm)	Automatic	11.33	197 ± 61
	Manual	9.04	157 ± 46
Double (13.5 cm)	Automatic	12.28	214 ± 66
	Manual	9.71	168 ± 50

mAs_mod_ corresponds to the mean of the modulated mAs during the entire examination.

**Figure 4:**
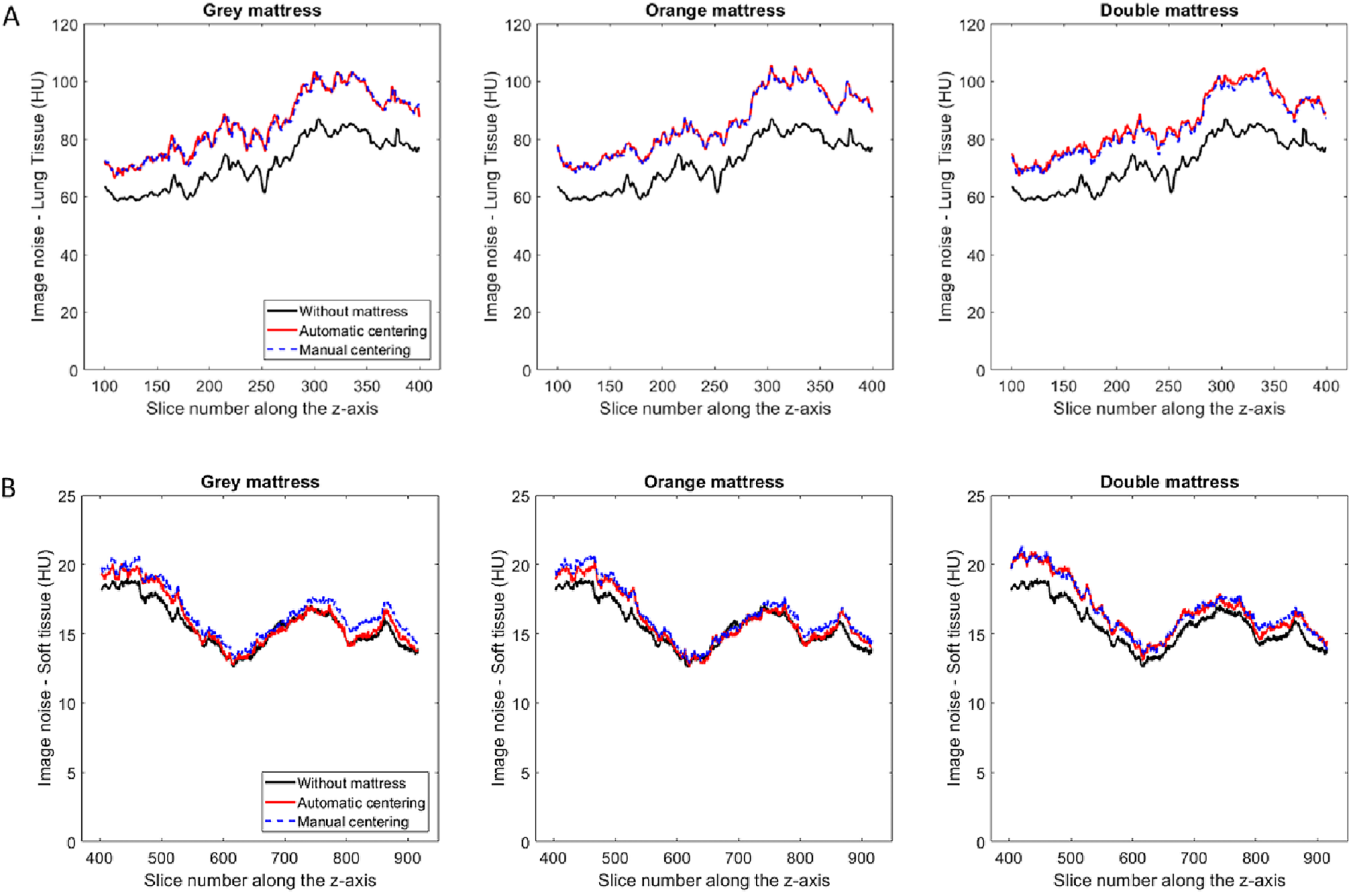
(**a**) Image noise along the z-axis obtained in the lung tissues in the chest area for image acquisitions with additional mattresses after automatic and manual phantom centering. (**b**) Image noise along the z-axis obtained in the soft tissues in the abdomen-pelvis area for image acquisitions with additional mattresses after automatic and manual phantom centering.

Figure 4 depicts the image noise measured along the z-axis for the lung tissues in the chest (Fig. 4a) and the soft tissues in the abdomen and-pelvis (Fig. 4b) for acquisitions with additional mattresses after automatic and manual phantom centering.

For each acquisition and both areas, the image noise was significantly higher using an additional mattress than without mattress (p < 0.05). For both areas and each additional mattress, image noise was significantly higher (p < 0.05) for automated phantom centering than with manual phantom centering (Table 3).

**Table 3:** Image noise obtained in the lung tissues in the chest area and in the soft tissues in the abdomen-pelvis area for acquisitions of phantom with and without an additional mattress.

Mattress	Patient centering	Lung tissues	Soft tissues
Without	Automatic	71.6 ± 8.8	15.7 ± 1.7
Grey (3.5 cm)	Automatic	85.3 ± 10.7	16.4 ± 1.9
	Manual	84.1 ± 10.7	16.0 ± 1.9
Orange (10 cm)	Automatic	85.4 ± 10.8	16.6 ± 2.0
	Manual	84.3 ± 10.7	16.1 ± 2.0
Double (13.5 cm)	Automatic	85.6 ± 10.5	16.8 ± 2.0
	Manual	84.4 ± 10.3	16.2 ± 2.0

## Discussion

For the first time, this study assessed the automated patient positioning proposed by a 3D camera for body contour detection with the use of additional mattresses. Although the 3D camera hasn’t been calibrated and approved for that, this is a situation encountered very often when acquiring CT scans in clinical practice in emergency departments. The results of this study show that the use of an additional mattress between the patient's back and the table top would not be correctly taken into account by the 3D camera, resulting in incorrect patient height positioning, as warned for in the user manual of the 3D-camera. This resulted in an increase in the dose delivered to the patient and higher image noise, especially for thicker mattresses. Manual correction of the patient’s centering by the radiographer is therefore essential when an additional mattress is used.

The outcomes found in this study show that the automated positioning proposed by the 3D camera places the patient above the CT isocentre when an additional mattress is used. This incorrect centering is related to the 3D camera algorithm failing to consider this additional thickness. Indeed, the 3D camera is calibrated to take into account the distance between the table top and the camera and deduce the thickness of the patient as a function of the distance between the top of the patient and the camera as measured on the initial image. The camera first proposes a fallback isocentre and then a more precise isocentre adjusted to the differences in the patient’s height along the z-axis in the area to be radiographed. With this additional mattress thickness, the patient height is increased, and the isocentre proposed by the camera is therefore placed above the isocentre of the CT scan. For orange and double mattresses, we found that overall, the camera proposed placing the table at a height of approximately half the mattress thickness above the ideal table position. This takes into account the compression of the mattress and the air space between the mattress and the table for the specific anatomical zone under consideration.

We also showed that this incorrect centering in terms of height leads to an increase in the dose delivered to the patient and in the image noise. In fact, we found that for thicker mattresses, the patient was placed at a greater distance above the CT scan isocentre, and the modulated mAs and image noise were increased further. Similar results regarding the impact of incorrect patient centering on dose and image noise have been published previously^6,10,11^. The increase in dose and image noise is related to how the bowtie filter and the TCM system work. Indeed, optimal functioning of these two elements requires that the patient be placed correctly at the isocentre^6,10–12^. The bowtie filter allows correction of the attenuation differences between the centre and the periphery of the patient to homogenize the signal received by the CT detectors. If the patient is off-centered, the bowtie filter correction would be non-uniform, resulting in an increase in the image noise and an incorrect dose distribution for the patient^6^. In addition, in the present study, anteroposterior topographic images were obtained before each acquisition. The higher the phantom was placed above the isocentre, the more magnified the phantom appeared on the topographic image, resulting in a higher mAs_mod_ as defined by the TCM along the z-axis. Similar outcomes were found by Kaasalainen et al. on chest CT for different phantom sizes^6^. We found that after placing the phantom at the isocentre, the mAs_mod_ and the image noise along the z-axis were reduced regardless of the mattress thickness. The decrease of image noise after manual centering were found for all acquisitions, confirming the efficiency of bowtie filter correction and the TCM system. However, we also found a significant difference in mAs_mod_ between the reference acquisition and the acquisition with the 13.5 cm mattress. This result was related to the increase in the additional attenuation of the mattress, which led to an increase in the mAs offered by the TCM system. Indeed, we found that the thicker the mattress was, the greater the increase in mAs_mod_ with respect to the reference acquisition. It should be noted that all these outcomes were obtained based on the anteroposterior topographic images. In clinical practice, posteroanterior topographic images are also used to minimize the dose to radiosensitive organs such as breasts or ovaries. Using these topographic images, the reverse situation would likely have occurred with a reduced phantom size in these images and a lower mAs_mod_ as defined by the TCM along the z-axis. In all cases and whatever the topographic images, the precise centering of the patient is a major issue in the quality of the examination.

In clinical practice, the use of 3D camera leads to real benefits to radiographers in terms of patient care. Under most circumstances and within the field of use defined by the manufacturer, the 3D camera developed by Siemens Healthineers allows the patient to be centered at the isocentre precisely, which improves examination practices and quality from a dosimetric and image quality point of view^7,8^. However, the manufacturer warns in the user manual explicitly that the 3D workflow is not optimized for examinations that use non-original and non-released equipment, such as the mattresses, because it can impact the detection of body landmarks or the patient’s isocentre. Therefore, when using an additional mattress, the radiographer can principally use the 3D-camera for the automated longitudinal patient centering proposed by the 3D camera but it is essential to check the vertical centering proposed and manually correct if necessary. An upfront manual vertical positioning in such cases is preferred though, to avoid potential risks of uncorrected positioning provided by the 3D camera in uncalibrated cases.

In the emergency department, the use of mattresses to facilitate the transfer of non-mobile patients and those in extreme pain or for polytrauma patients requires evolving the algorithm of the 3D camera. A simple solution would be to enter the weight and height of the patient and the thickness of the mattress so that the algorithm can apply a corrective factor taking into account the type of mattress and its compressibility. This solution would, however, not be very applicable for polytrauma patients, who are placed most often on the CT scan table with a shell mattress and medical equipment. In all cases, the use of a mattress should be limited because a mattress induces additional attenuation, which increases the dose delivered to the patient. An additional mattress should only be used if the patient is unable to move or is in extreme pain, in which case it should be as thin and little attenuating as possible. In our institution, we will ask that the use of the grey 3.5 cm mattress be generalized to all stretchers. It is also essential that radiographers become aware of the impact of routine mattress use. Last, as the majority of patients were transferred to the CT table with no additional mattress our results should not impact much the positioning management of the patients in emergency CT practice.

This study has limitations. First, this study was evaluated on a single CT system. However, the methodology applied can be transferred to other CT systems equipped with the same 3D camera. Second, we carried out this study with the three most commonly used mattresses, but other mattresses can occasionally be used. However, the ones used in this study represent the thickest and thinnest mattresses routinely used in our institution. Then, only one acquisition was performed per mattress but the reproducibility of the tube current modulation system on phantoms has previously been assessed^13^. Finally, this study was carried out only on a single anthropomorphic phantom with a relatively weak morphology. However, the results would have been appreciably equivalent to those with phantoms of stronger morphology. Additional studies on patients should be performed to confirm these results.

## Conclusion

This study confirmed that the automatic vertical centering proposed by the 3D camera was incorrect when an additional mattress was positioned between the table and the patient, as warned for by the manufacturer. Off-centering of the patient above the isocentre leads to an increase in the dose delivered and higher image noise. Manual correction of this vertical centering proposed by the 3D camera, or direct manual centering by the radiographer is therefore essential when an additional mattress is used. An improvement in the 3D camera algorithm is desirable, particularly for CT scanners placed in emergency departments where mattresses are regularly used. Further patient studies could be conducted to confirm the outcomes obtained with phantoms.

## Abbreviations

CT: Computed tomography
CTDI_vol_: Volume CT dose index
DICOM: Digital imaging and communications in medicine
mAs_mod_: Modulated mAs
RDMS: Radiation dose management system
TCM: Tube current modulation
